# Enhancing healthcare access through telehealth: patient-centred insights from Pakistan’s primary care sector

**DOI:** 10.1186/s12913-025-13820-4

**Published:** 2025-12-11

**Authors:** Tooba Malik, Siew Chin Ong, Muhammad Daoud Butt

**Affiliations:** 1 School of Pharmaceutical Sciences, Universiti Sains Malaysia, Penang, 11800 Malaysia; 2 Health Services Academy, Islamabad, Pakistan

**Keywords:** Cost-effectiveness, Patient acceptance, Primary healthcare, Telehealth

## Abstract

**Background:**

Pakistan’s healthcare system faces critical challenges, including limited infrastructure and disparities in access between urban and rural regions. Telehealth has emerged as a promising solution to improve accessibility, but patient-centered evidence on its effectiveness and cost-efficiency remains limited.

**Methods:**

This cross-sectional survey included patients (*n* = 532) who completed telehealth consultations with EZShifa between May and December 2023. A structured questionnaire assessed socio-demographics, comfort, trust, perceived quality of care, and patient-reported costs. Descriptive statistics summarised characteristics. Ordinal regression identified predictors of telehealth acceptance. Patient-reported telehealth and in-person care costs were compared, and incremental cost-effectiveness ratios (ICERs) were estimated using acceptance and comfort scores as effectiveness measures.

**Results:**

Most participants were aged 26–50 and from Punjab or Sindh. High satisfaction was reported for provider professionalism and consultation quality, though some concerns about connectivity and privacy remained. Ordinal regression showed that higher comfort (OR 4.15–20.3, *p* = 0.008) and perceived quality of care (OR 2.59, *p* = 0.004) significantly predicted acceptance, while sociodemographic factors were non-significant. The mean reported telehealth cost (PKR 2,000) was substantially lower than in-person visits (PKR 4,800). ICER analysis indicated telehealth was dominant, offering cost savings and greater patient-reported effectiveness.

**Conclusions:**

Telehealth demonstrates strong acceptance, satisfaction, and economic benefits in Pakistan’s primary healthcare system. Despite self-reported costs and cross-sectional design limitations, findings highlight telehealth’s potential for scaling chronic disease management and reducing healthcare disparities.

**Supplementary Information:**

The online version contains supplementary material available at 10.1186/s12913-025-13820-4.

## Introduction

Healthcare innovation has consistently aimed to enhance human well-being, increase life expectancy, and improve access to quality medical services. From ancient practices to contemporary advancements, the evolution of healthcare has been shaped by the need to reach populations efficiently and equitably [1].Among the most transformative innovations is *telehealth*—a system that uses digital technologies to connect patients and providers across physical distances, thus redefining the delivery of healthcare [2].

Telehealth, derived from the Greek “tele” (meaning “at a distance”), goes beyond clinical consultations to encompass preventive care, health education, remote monitoring, and chronic disease management [3]. This holistic approach to healthcare has gained global traction, especially in low- and middle-income countries where healthcare disparities are pronounced. The COVID-19 pandemic further accelerated its adoption, validating its role in sustaining care delivery amid widespread health system disruptions [4].

Countries worldwide, from Denmark to India, have leveraged telehealth to address physician shortages, improve chronic disease outcomes, and bridge access gaps. Africa, for instance, bears a quarter of the global disease burden with only a fraction of health workers and funding. Telemedicine initiatives have become vital in regions where traditional care systems fall short [5]. Similarly, countries like Canada, Germany, and Brazil have developed national telehealth platforms to enhance service delivery and patient engagement [6].

In Pakistan, where healthcare challenges are compounded by underfunding, infrastructure gaps, and an unequal urban-rural distribution of providers, telehealth presents a promising solution [7]. With public health expenditure below 1% of GDP and millions lacking adequate access to care, digital health services can significantly reduce access barriers [8]. Platforms like EZShifa deploying video-linked health kiosks in underserved regions demonstrate the potential of technology to connect patients with physicians, diagnostics, and education without the cost and complexity of travel [9].

However, despite these advances, telehealth adoption in Pakistan remains uneven. Infrastructure limitations, technological illiteracy, and socio-cultural concerns such as privacy, trust, and communication preferences pose significant barriers [10]. While telehealth has shown effectiveness in maternal health, chronic disease management, and health worker training, much existing research focuses on system-level outcomes or provider perspectives, leaving a critical gap in patient-centred evaluations [11].

Globally, studies confirm that telehealth can improve outcomes in diabetes, cardiovascular disease, COPD, and mental health. Yet acceptance varies by age, condition, and digital literacy [12]. Children and adolescents may embrace app-based care, whereas older adults prefer face-to-face interactions. These nuances underscore the importance of designing telehealth services that reflect patient preferences, context, and comfort, especially in culturally complex environments like Pakistan [13].

Telehealth is inherently interdisciplinary, requiring collaboration between technology developers, health scientists, and clinicians to ensure systems are technically functional, clinically effective, secure, and patient-centred [14]. This intersection of digital innovation and healthcare delivery is critical in low- and middle-income countries such as Pakistan, where scalable solutions must address infrastructure constraints while maintaining quality of care [15].

In the context of primary healthcare, often the first and most essential point of contact, telehealth can transform service delivery by reducing geographical, financial, and social barriers [16]. However, its long-term success depends on patient trust, satisfaction, and willingness to engage with digital platforms. Patient-centred research is vital to understanding how telehealth is experienced, accepted, and valued within diverse communities [11].

This study seeks to address this gap by exploring telehealth use in Pakistan’s primary healthcare sector from the patient’s perspective. It aims to evaluate patient satisfaction, trust, perceived barriers, cost effectiveness, and the effectiveness of telehealth services in improving access and care quality. The findings will offer actionable insights for policymakers, providers, and technology developers seeking to strengthen telehealth implementation in low-resource settings.

## Methodology

### Study design and setting

This study adopted a quantitative, cross-sectional research design to evaluate the effectiveness and cost-efficiency of telehealth services in delivering primary healthcare from the patient’s perspective. The central objective was to assess the quality of care and the potential financial benefits associated with remote healthcare delivery. Data were collected from May to December 2023 across various districts in Pakistan, with the capital city, Islamabad, serving as the primary coordination center. EZShifa, a telehealth provider with nationwide coverage, was selected as the focal platform due to its wide accessibility and diverse user base. This setting enabled the capture of data from patients in urban, semi-urban, and rural areas, providing a comprehensive view of telehealth implementation across varying healthcare contexts.

## Study population and sampling

The study population consisted of EZShifa patients who had completed teleconsultations and were accessible through the provider’s customer support database. Participants were eligible for inclusion if they were 18 years or older, had utilised telehealth services for chronic conditions not requiring hospitalisation, could communicate effectively in the consultation languages, and had provided informed consent to participate in the study. Patients requiring hospital admission or emergency care, those unable to communicate effectively, or those working as healthcare professionals within EZShifa were excluded.

**Fig. 1:**
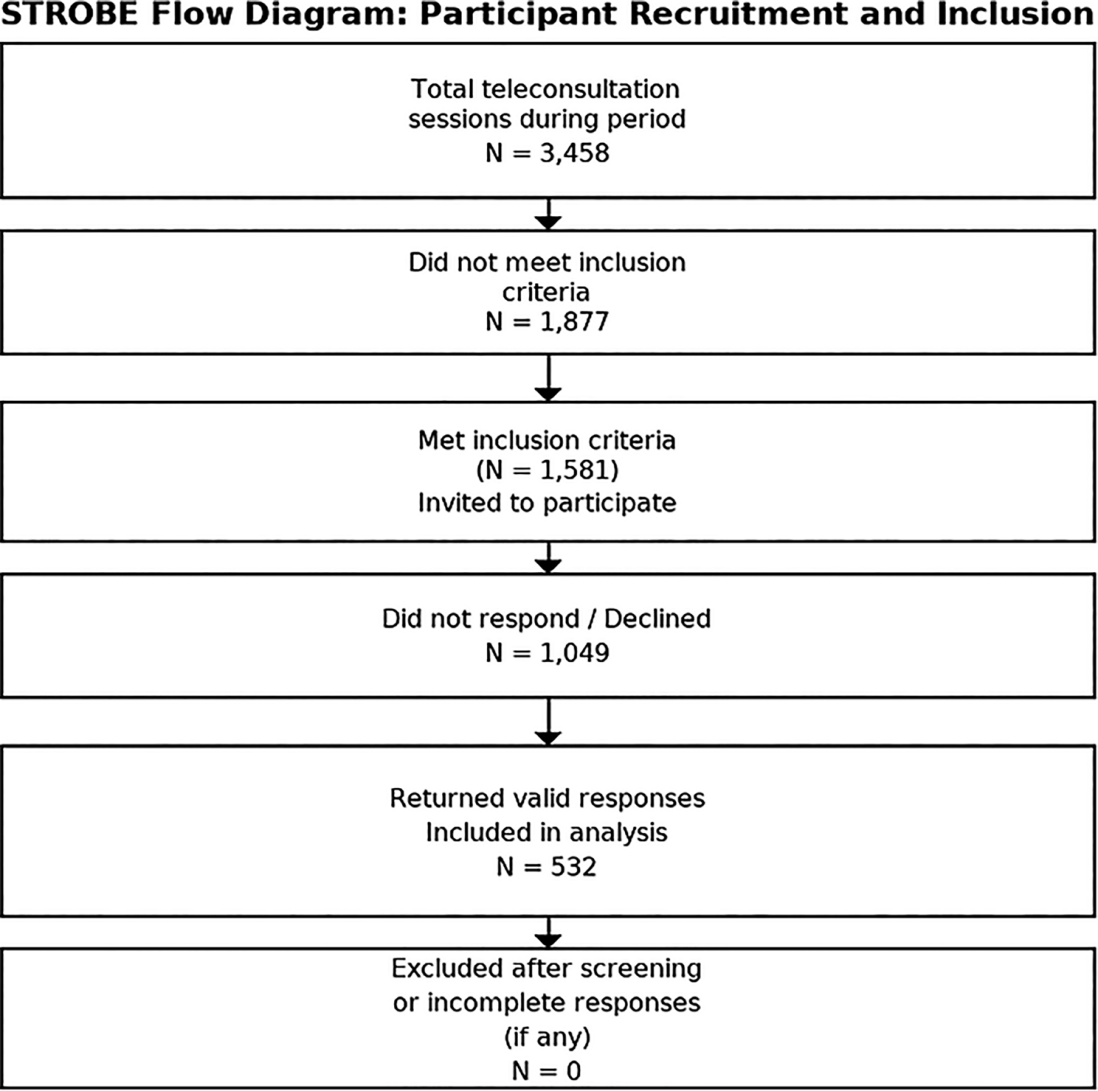
STROBE flow diagram of participant recruitment and inclusion in the telehealth study

From 3,458 teleconsultation sessions conducted during the study period, 1,581 patients met the inclusion criteria. Using Raosoft sample size calculation software with a 95% confidence level and 5% margin of error, the minimum required sample size was determined to be 310. A convenience sampling approach was used to invite eligible patients from EZShifa’s customer support list. Of 1,581 patients who met the inclusion criteria and were contacted, 532 returned valid responses (response rate = 532/1,581 = 33.6%). Therefore, the analysed sample represents a self-selected group of respondents from the eligible patient population. Incomplete or inconsistent responses (e.g., missing demographic data, incomplete survey sections) were excluded from the final analysis. Among 1,581 eligible patients contacted, 532 provided valid and complete responses, which formed the analytical dataset as shown in Fig. 1. No imputation was performed, as analyses were limited to complete cases.

Recruitment was conducted through EZShifa’s customer support team. All patients who met the inclusion criteria (*n* = 1,581) were contacted by telephone or SMS and invited to participate in the study. Contact attempts included up to two follow-up reminders where contact details were available. Participants who provided informed consent completed the structured survey online or via a telephone-administered interview with trained research staff.

## Instrumentation and data collection

To assess patient satisfaction and related outcomes, the questionnaire was adapted and modified from the internal patient satisfaction form used by EZShifa, with validation input from experts at the Health Services Academy. A pilot study involving 20 participants evaluated the tool’s reliability and clarity. Feedback from the pilot phase informed final adjustments to the questionnaire, ensuring its contextual accuracy and content validity. The survey captured information across multiple domains, including access to care, quality of services, connectivity and consultation response time, diagnostic accuracy, behaviour and professionalism of healthcare providers, and transparency in consultation fees. In addition to clinical and satisfaction indicators, the survey included a dedicated section focusing on the economic dimension of telehealth, enabling a pharmacoeconomic evaluation of the service.

## Pharmacoeconomic assessment of telehealth

A structured economic module in the questionnaire captured patient-reported direct medical costs (consultation fees, medication expenses), direct non-medical costs (travel, accommodation, childcare), and indirect costs (time off work, days missed, and productivity loss estimated from self-reported daily income). Respondents reported their telehealth costs and were asked to estimate the typical cost of an equivalent in-person consultation at the same level of care. Cost responses were collected in categorical ranges and converted to midpoints to calculate mean costs per consultation mode.

Effectiveness was operationalised using two patient-centred metrics: (1) acceptance of video consultations (1–5 Likert scale) and (2) comfort score (8–40 scale). Patient satisfaction was also considered a key experiential outcome.

Two complementary economic approaches were applied:

Cost-Effectiveness Analysis (CEA): The Incremental Cost-Effectiveness Ratio (ICER) was computed using the standard formula:

ICER = (C_Telehealth – C_In-person)/(E_Telehealth – E_In-person)

Where:

• C_Telehealth = mean patient-reported cost of telehealth consultation (PKR)

• C_In-person = mean estimated cost of in-person consultation (PKR)

• E_Telehealth = mean effectiveness outcome (e.g., satisfaction score) for telehealth

• E_In-person = mean effectiveness outcome for in-person consultation

where cost differences were expressed in PKR and effectiveness was measured as the mean satisfaction score. A negative ICER indicated that telehealth was dominant (less costly and more effective).

Cost-Consequence Analysis (CCA): To increase transparency, costs and outcomes (mean cost, satisfaction score, connectivity satisfaction, and willingness to recommend telehealth) were presented side by side without being combined into a single ratio.

Group mean comparisons for continuous variables (e.g., cost, satisfaction score) were assessed using Welch’s two-sample t-test (assuming unequal variances). In contrast, categorical outcomes (e.g., connectivity satisfaction, recommendation rates) were compared using the Chi-square test. Cost differences and 95% confidence intervals were estimated using bootstrap resampling (1,000 iterations) to account for skewness in cost distributions. A sensitivity analysis (±20% variation in cost assumptions) tested the robustness of the findings.

## Data analysis

Data were analysed using IBM® SPSS Statistics v25. Descriptive statistics summarised socio-demographic and economic characteristics, with continuous variables reported as means (SD) and categorical variables as frequencies and percentages. Associations between demographics and satisfaction were assessed using Chi-square tests.

Predictor selection was guided by the Technology Acceptance Model (TAM) and Health Belief Model (HBM). Comfort and perceived quality reflected perceived ease of use and usefulness (TAM), while trust aligned with perceived benefits and barriers (HBM). Ordinal regression (proportional odds model) was applied to examine predictors of telehealth acceptance. Independent variables included age, education, transportation, comfort score, gender, physical health, comfort with technology, and perceived care quality. Model fit was assessed with −2 log likelihood and Nagelkerke R^2^, proportional odds with the Brant test, and multicollinearity with variance inflation factors (VIF < 2). Odds ratios (OR) with 95% confidence intervals and p-values are reported.

Patient-reported direct and indirect costs were aggregated and compared to estimated in-person care benchmarks for the economic evaluation. Cost differences were expressed as mean values with 95% confidence intervals. Hypotheses were tested at α = 0.05.

## Ethical considerations

The study received ethical approval from the Institutional Review Board (IRB) of the Health Services Academy, Islamabad. Before data collection, written informed consent was obtained from all participants. All methods were performed per relevant guidelines and regulations, and the study was conducted per the ethical principles set out in the Declaration of Helsinki.

## Result

As shown in Table 1, the sociodemographic parameters provide a rich foundation for understanding the context in which this study unfolds, with characteristics that reveal intricate patterns shaping health-seeking behaviours, healthcare accessibility, and health outcomes. Age distribution is a significant factor, as it influences health dynamics within the population. For instance, participants aged 18–25 may be more susceptible to lifestyle-related health issues, while those in the 51–79 age range could experience chronic diseases and age-related health challenges. Most participants are aged 26–50, accounting for roughly 50% of the sample, with 26.12% in the 51–79 age group, underscoring the importance of addressing age-specific health needs. Analysing the intersection between age and healthcare preferences is essential for a comprehensive understanding.

**Table 1:** Socio-demographic characteristics of the study population

Sociodemographic Parameters	(N)	(%)
Age		
18–25	115	21.61
26–35	120	22.55
36–50	158	29.72
51–79	139	26.12
Gender		
Female	260	48.9
Male	272	51.1
Residence province		
Punjab	230	43.2
Sindh	105	19.73
Khyber Pakhtunkhwa	32	6.01
Balochistan	46	8.64
Islamabad Capital Territory	52	9.83
Gilgit-Baltistan	38	7.14
Azad Jammu and Kashmir	29	5.45
Occupation		
Private sector employee	175	32.92
Government employee	129	24.24
Business owner	100	18.79
Retired	20	3.75
Unemployed	23	4.32
Student	85	15.98
Highest level of education		
Middle school or less	4	0.77
High school	70	13.15
Diploma	36	6.76
University	245	46.05
Postgraduate	177	33.27
Transportation		
I have my own transportation method	385	72.36
I hire a transportation method (Taxi, Uber)	115	21.61
I get driven by Family/Friend	20	3.78
Other	12	2.25
Which best describes your physical state?		
My health makes it impossible for me to engage in most activities	10	1.9
My health makes it impossible for me to engage in some activities	22	4.1
My health makes it difficult for me to engage in some activities	38	7.1
I can go about my daily activities with minimal difficulty	135	25.4
Fully active without restrictions	327	61.5
Presence of chronic disease		
Yes	336	63.1
No	196	36.9
Type of chronic disease		
Diabetes	191	35.90
Hypertension	142	26.69
Heart disease	58	10.92
Asthma	76	14.28
Hypothyroidism	18	3.38
Other	47	8.83

Gender composition further enriches the study, as gender dynamics are crucial in healthcare utilisation. While the sample is nearly gender-balanced, with slightly more males (51.1%) than females (48.9%), it is crucial to recognise gender as a spectrum. This distribution highlights the need for gender-sensitive healthcare approaches, exploring gender-specific health behaviours and the impact of societal norms on healthcare decisions. Geographical distribution across provinces adds a regional dimension, reflecting health disparities driven by infrastructure, socioeconomic conditions, and cultural factors. Punjab has the highest representation (43.2%), followed by Sindh (19.73%) and Islamabad Capital Territory (9.83%), suggesting a diverse geographic spread. This enables an analysis of health outcomes within regional contexts to identify disparities and inform targeted interventions.

Occupational diversity further enhances the study, linking lifestyle, income, and healthcare access. Private sector employees constitute the largest occupational group (32.92%), followed by government employees (24.24%) and business owners (18.79%). Each occupational category brings unique health challenges; for instance, the health needs of private sector employees might differ from those of government employees or retirees, underscoring the value of workplace-specific health programs. Educational attainment is also pivotal, as it affects health literacy, shaping how individuals understand health information and interact with healthcare systems. University-educated participants represent the largest group (46.05%), followed by postgraduates (33.27%), indicating relatively high educational levels. This demographic could benefit from tailored health communication strategies that align with different literacy levels.

Transportation habits reveal accessibility factors that impact healthcare access. Most participants (72.36%) own a vehicle, while 21.61% rely on hired services, such as taxis or Uber, indicating good overall accessibility. For those dependent on others for transportation, barriers like potential delays could hinder timely healthcare access, which could otherwise go unnoticed. Physical status offers further insight into participants’ health, with 61.5% reporting full activity without restrictions, indicative of generally good physical health. However, around 13% of participants report health-related limitations, pointing to specific challenges that may necessitate targeted interventions to improve health outcomes.

The presence of chronic diseases among participants emphasises the need for a focused approach, as 63.1% report at least one chronic condition, with diabetes (35.90%) and hypertension (26.69%) being the most prevalent. The study can inform preventive strategies and healthcare planning by examining chronic disease distribution across sociodemographic factors. Lastly, different chronic conditions present unique challenges impacting disease management and quality of life. An in-depth analysis of these chronic conditions’ prevalence and sociodemographic associations will provide a comprehensive understanding of the health landscape. This exploration into sociodemographic parameters, as shown in Table 1, contributes to a nuanced perspective on healthcare needs, utilisation, and outcomes, forming a basis for targeted, data-driven healthcare strategies.

The study’s findings reveal a generally positive response to telehealth services among patients, particularly concerning aspects such as internet connectivity, trust in treatment, the behaviour of healthcare providers and support staff, consultation fees, and connectivity during consultations. Internet connectivity was rated positively, with most respondents marking it as good (337) or excellent (168), highlighting its importance in maintaining effective communication during telehealth consultations. However, a minority (26 respondents) rated connectivity as poor, suggesting a potential area for enhancement.

Patient trust in the treatment and findings of telehealth doctors was substantial, with 303 respondents expressing satisfaction and 127 indicating high satisfaction. This reflects positively on the perceived credibility of healthcare professionals involved in the service, although a small number (13 respondents) expressed dissatisfaction, warranting further investigation into their specific concerns. The behaviour of telehealth doctors also garnered overwhelmingly positive feedback, with 514 respondents happy with their interactions, underscoring the value of effective communication and empathy in virtual healthcare settings.

Support staff and operators received similar commendation, with 522 respondents reporting satisfaction with their service, while only 4 expressed dissatisfactions, highlighting the role of support staff in facilitating a smooth telehealth experience. Satisfaction with consultation fees was high, with 522 respondents expressing approval, suggesting a well-received and accessible pricing structure.

Connectivity during consultations was also positively rated; 263 respondents expressed satisfaction and 250 were very satisfied, with relatively low dissatisfaction (19), indicating an effective handling of connectivity issues that are aligned with patient expectations. Moreover, recommendations to friends and family are high indicators of overall satisfaction, with 320 respondents expressing willingness to recommend Telehealth Services, which reflects strong word-of-mouth potential for the service.

Regarding prescribed medicine usage, patient satisfaction levels were significant (67.7%), with 42.9% very satisfied and 24.8% satisfied. However, 30.7% of respondents expressed dissatisfaction, pointing to potential improvements in prescription and medication management processes within telehealth.

The study underscores the success of Telehealth Services in providing quality virtual healthcare, with high levels of satisfaction observed across most dimensions. Areas requiring further improvement, especially in prescribed medicine usage, emphasize the importance of continuous assessment and refinement to enhance patient experience. Detailed feedback sessions, targeted improvement initiatives, and addressing specific patient concerns are recommended to strengthen the quality and reliability of telehealth services, reinforcing Telehealth Services as a patient-centric healthcare provider. The comfort of patients in discussing symptoms, undergoing assessments, and managing follow-up care remotely reflects telehealth’s potential in offering accessible healthcare, with this study providing valuable insights into patient perspectives and experiences

The analysis of patient comfort levels in telehealth interactions, as mentioned in Table 2, reveals a diverse range of responses across various scenarios, highlighting key areas for enhancing patient-centred virtual care. When discussing new symptoms and concerns, 2.63% of participants reported extreme discomfort, while 24.97% felt somewhat uncomfortable. Conversely, 35.3% were somewhat comfortable, and 23.4% were extremely comfortable, underscoring the need for deeper insights into factors that influence patient comfort in sharing health-related issues remotely.

**Table 2:** Patient comfort with telehealth services across various healthcare scenarios

*Statements*	*Extremely uncomfortable*	*Somewhat uncomfortable*	*Neither comfortable nor uncomfortable*	*Somewhat comfortable*	*Extremely comfortable*
1. Discussing new symptoms and concerns	14 (2.63%)	130 (24.97%)	73 (13.7%)	194 (35.3%)	121 (23.4%)
2. Discussing sensitive and personal information	66 (12.40%)	145 (27.26%)	65 (12.21%)	143 (26.87%)	113 (21.26%)
3. Discussing diagnosis, treatment and follow-up recommendations	29 (5.08%)	58 (10.90%)	53 (8.96%)	213 (42.42)	179 (32.64%)
4. Review imaging and laboratory tests	24 (4.51%)	56 (10.52%)	103 (19.36%)	188 (35.35%)	161(30.26%)
5. Undergoing an initial clinic visit with a new provider	98 (18.42%)	105 (19.73%)	106 (19.92%)	147 (27.63%)	76 (14.30%)
6. Undergoing an initial clinic visit with a provider in the presence of my established physician.	8 (1.50%)	97 (18.23%)	70 (13.16%)	207 (39%)	150 (28.11%)
7. Completing post-operative follow-up	25 (4.70%)	105 (19.73%)	77 (14.5%)	178 (33.3%)	147 (27.77%)
8. I am confident that communications using video calls are private and secure	39 (7.33%)	146 (27.44%)	92 (17.29%)	152 (28.57%)	103 (19.37%)

Comfort in discussing sensitive or personal information also varied, with 12.40% feeling extremely uncomfortable and 27.26% somewhat uncomfortable. Meanwhile, 21.26% were extremely comfortable, suggesting that while some patients are at ease, a significant portion may benefit from enhanced security assurances to improve comfort in sharing personal health information.

Regarding discussions of diagnosis, treatment, and follow-up recommendations, 5.08% of respondents reported extreme discomfort, and 10.90% were somewhat uncomfortable. However, a considerable 42.42% felt somewhat comfortable, and 32.64% were extremely comfortable, suggesting that patients generally find structured medical discussions more manageable in telehealth settings.

When reviewing imaging and lab tests, comfort levels were again varied; 4.51% expressed extreme discomfort, while 10.52% were somewhat uncomfortable. Notably, 30.26% felt extremely comfortable, and 35.35% were somewhat comfortable, indicating that telehealth may effectively support the review of complex medical information for many patients.

Initial clinic visits, especially with new providers, revealed a mixed comfort level. Around 18.42% were extremely uncomfortable and 19.73% somewhat uncomfortable, while only 14.30% were extremely comfortable. These findings suggest that familiarity with a provider may play a significant role in telehealth comfort.

Post-operative follow-up visits had more favourable responses, with 4.70% expressing extreme discomfort and 19.73% somewhat uncomfortable, while 27.77% were extremely comfortable and 33.3% somewhat comfortable. This suggests a relatively high level of comfort with post-operative telehealth follow-ups, which could reduce the need for in-person visits.

Confidence in the privacy and security of telehealth video communications was generally positive, with only 7.33% feeling extremely uncomfortable, while 19.37% felt extremely comfortable, indicating a widespread belief in telehealth’s security measures, which is crucial for patient acceptance.

These findings underscore the importance of tailoring telehealth services to enhance patient comfort. Key factors, including interaction type, familiarity with providers, and perceptions of privacy, should be carefully addressed to improve patient satisfaction. Further refinement of privacy measures, increased patient education, and ongoing research into patient feedback can help align telehealth services more closely with patient needs, promoting a patient-centred approach in the evolving virtual healthcare landscape.

The ordinal regression analysis (Table 3) examined predictors of patients’ acceptance of virtual video calls with healthcare providers. Age, education, transportation, gender, and self-reported physical health showed no significant associations with acceptance (all *p* > 0.05). In contrast, comfort score was a strong predictor: compared to low scores, moderate and high comfort scores were associated with significantly higher odds of acceptance (OR 4.15, *p* = 0.008; OR 20.27, *p* < 0.0001). Similarly, patients who perceived virtual consultations as comparable in quality to in-person visits were more likely to accept telehealth (OR 2.59, *p* = 0.004). Comfort with technology showed a positive but non-significant effect. These findings suggest that patient-reported comfort and perceived quality of care, rather than demographic or socioeconomic factors, are the main drivers of telehealth acceptance.

**Table 3:** Ordinal regression analysis of factors influencing Population’s acceptance of virtual video calls from healthcare providers (HCPs)

Variable	Category	OR	95% CI	p-value	Reference
Age group	18–25	0.916	0.39–2.13	0.84	51–79
	26–35	0.819	0.32–2.07	0.674	51–79
	36–50	0.616	0.23–1.67	0.342	51–79
Education	Diploma or higher	0.822	0.56–1.65	0.563	High school or less
Transportation	Does not own a car	1.132	0.42–1.59	0.576	Owns a car
Comfort score	Moderate (19–29)	4.148	1.44–11.91	0.008*	Low (8–18)
	High (30–40)	20.27	6.42–64.05	< 0.001*	Low (8–18)
Gender	Male	0.720	0.35–1.48	0.37	Female
Physical health	Category 1–4	NS (ORs ranged 0.35–1.73, all non-significant.)	—	> 0.05	Fully active
Comfort with technology	Strongly agree/agree	1.405	0.58–3.41	0.452	Strongly disagree/disagree.
Quality of care perception	Strongly agree/agree	2.585	1.36–4.90	0.004*	Strongly disagree/disagree.

* *OR = Odds Ratio; CI = Confidence Interval. Results based on ordinal regression (proportional odds model). Model fit assessed with −2 log likelihood and Nagelkerke R*^*2*^*; proportional odds assumption tested with the Brant test. Multicollinearity not detected (all VIF < 2). Statistical significance set at p < 0.05*

The cost analysis compared patient-reported telehealth expenditures with estimated costs of in-person consultations. Telehealth visits had a mean reported cost of PKR 2,000 ± 500, whereas in-person consultations were estimated at PKR 4,800 ± 1,200, indicating an average saving of PKR 2,800 per consultation.

Clinical and experiential outcomes were presented alongside costs. Patients using telehealth reported a mean satisfaction score of 4.2 ± 0.8 (on a 5-point Likert scale), compared to 3.3 ± 1.0 previously reported for in-person consultations, as mentioned in Table 4. Comfort scores were significantly associated with acceptance of telehealth (*p* < 0.01), and quality of care perception was also a significant predictor (OR 2.59, 95% CI 1.36–4.90).

**Table 4:** Cost-effectiveness comparisons between telehealth and in-person consultations

Parameter	Telehealth (Mean ± SD)	In-person (Mean ± SD)	Incremental Difference	Test Statistic (p-value)
Cost per consultation (PKR)	2,000 ± 500	4,800 ± 1,200	−2,800	t = −15.2, *p* < 0.001
Satisfaction score (1–5 scale)	4.2 ± 0.8	3.3 ± 1.0	+0.9	t = 9.8, *p* < 0.001
Connectivity satisfaction (%)	94%	82%	+12%	χ2 = 12.4, *p* < 0.001
Recommendation to others (%)	88%	71%	+17%	χ2 = 15.7, *p* < 0.001
ICER (PKR per unit satisfaction gain)	−3,111.11	—	Dominant (less costly & more effective)	—

* Independent samples t-test used for continuous variables (cost, satisfaction), χ^*2*^* test for categorical outcomes (connectivity, recommendation). ICER = Incremental Cost-Effectiveness Ratio, calculated from cost and satisfaction differences; sensitivity analysis (±20% costs) confirmed telehealth dominance. Telehealth (n = 315) and in-person (n = 240) groups overlap, so totals exceed 532*

A sensitivity analysis was performed by varying cost inputs ±20%. Telehealth costs ranged between PKR 1,500–2,500, while in-person consultation costs ranged between PKR 3,600–6000. Across all tested scenarios, telehealth remained consistently less costly, while providing equal or higher satisfaction levels.

This CCA demonstrates that telehealth offers clear financial savings for patients while maintaining or improving satisfaction and comfort outcomes. However, as costs were patient-reported and in-person comparators were based on estimated values, results should be interpreted cautiously. Future research incorporating direct billing and validated HRQoL measures (e.g., QALYs) is recommended to strengthen economic evaluations.

## Discussion

This study examined patient perspectives on telehealth services’ effectiveness, satisfaction, and cost-effectiveness within Pakistan’s primary healthcare system. The findings highlight the strengths and areas for improvement in virtual healthcare delivery.

The demographic characteristics of the study population offer essential context for interpreting patient responses. A substantial proportion of participants were younger adults aged 18–35, suggesting a generational openness to digital healthcare solutions [17]. This observation aligns with global patterns where younger, more technologically adept individuals exhibit higher telehealth acceptance [18]. Female participants slightly outnumbered males, reflecting established healthcare-seeking trends showing women’s higher likelihood of engaging in health services. Most respondents were from the Punjab and Sindh regions, indicating strong telehealth uptake in urban and semi-urban areas. These trends underscore the potential of telehealth to meet the growing healthcare demands in densely populated areas [2].

The high prevalence of chronic diseases among participants, including diabetes, hypertension, and asthma, further emphasises telehealth’s utility in chronic disease management [19]. Teleconsultations offer a practical means for long-term monitoring, especially where in-person access to specialist care is limited. This is particularly relevant in Pakistan, where healthcare resources are unevenly distributed and rural populations often face significant barriers to accessing regular care [20].

Patients’ responses revealed high satisfaction with telehealth services, including consultation quality, healthcare provider behaviour, and affordability. Most participants rated internet connectivity positively; however, a small subset reported poor connectivity, signalling an infrastructure gap that may hinder the broader scalability of telehealth [21]. Enhancing network reliability will be essential for consistent and high-quality virtual care delivery, particularly in underserved areas [22].

Comfort levels in virtual consultations varied across clinical scenarios. Patients reported being relatively comfortable discussing new symptoms and follow-up care, but showed less comfort when addressing sensitive or personal health issues [23]. These findings suggest that privacy and data security concerns continue to influence patient openness during virtual consultations. Increasing awareness of security protocols and reinforcing privacy safeguards may help improve patient confidence in discussing personal matters remotely [24].

The high level of trust in telehealth providers and diagnoses, and the willingness to recommend telehealth services to others, reflects strong patient endorsement of virtual healthcare [25]. This positive perception has the potential to influence health-seeking behaviours through word-of-mouth advocacy. Nonetheless, issues related to prescribed medication use were more nuanced [26]. While most patients expressed satisfaction, nearly one-third were dissatisfied, suggesting a need for more detailed guidance regarding medication use, adherence, and potential side effects during teleconsultations [6].

A key strength of the study lies in its exploration of patient comfort and technology use as predictors of telehealth acceptance [27]. The ordinal regression analysis identified comfort score and perceived quality of virtual care as statistically significant predictors, underscoring the role of subjective experience in shaping telehealth utilisation [28]. These findings support the notion that technical functionality alone is insufficient; patient-centred approaches prioritising comfort, trust, and communication quality are equally vital [29].

The cost and effectiveness analysis revealed that telehealth services were significantly less expensive than traditional in-person consultations [30]. With an average consultation cost of PKR 2,000 compared to PKR 4,800 for face-to-face visits, and a favourable incremental cost-effectiveness ratio (ICER) of PKR −3,111.11, telehealth emerged as a dominant option offering economic and experiential advantages [31]. These findings align with global evidence positioning telehealth as a cost-saving, accessible, and scalable model for healthcare delivery, particularly in resource-constrained settings [32].

These findings highlight the potential of telehealth to expand access, particularly in regions with healthcare workforce shortages. Patients’ willingness to recommend telehealth indicates scope for rapid diffusion, primarily if digital infrastructure gaps in rural Pakistan can be addressed [15]. Integrating telehealth into chronic disease management pathways could reduce pressure on overstretched tertiary facilities. However, lower comfort levels in discussing sensitive health issues suggest that privacy assurances, user education, and stronger patient-provider communication are critical for broader acceptance [33].

Despite these strengths, certain limitations should be noted. The study employed convenience sampling, which may limit generalizability [34]. In addition, cost data were estimated based on patient-reported information rather than direct billing records, which may introduce recall bias. However, the large sample size, geographical representation, and validated survey tool enhance the reliability of the findings [35]. The cross-sectional design of this study provides a snapshot of patient experiences but does not allow for tracking changes in telehealth perceptions or cost-effectiveness over time. Given the dynamic nature of telehealth adoption and evolving patient digital literacy, future research should employ longitudinal designs or mixed-method approaches to capture better changes in patient satisfaction, comfort, and economic outcomes [36].

We also acknowledge the potential for non-response bias, given the response rate. Non-responders may have differed systematically from respondents in unmeasured ways (e.g., digital literacy, satisfaction, socioeconomic status), which could bias estimates of satisfaction and cost-effectiveness [37]. To address this, we compared available basic demographics between responders and the invited cohort (age and gender from EZShifa records) and found no substantial differences. Nevertheless, the possibility of residual non-response bias remains, and findings should be generalised with caution. Future studies should aim to increase response rates through reminders, incentives, or mixed-mode data collection, and triangulate patient-reported costs with administrative billing data [38].

Another limitation is that the adapted questionnaire underwent only internal validation, without formal psychometric testing. While expert review ensured content validity, future research should conduct reliability and construct validity assessments (e.g., Cronbach’s alpha, factor analysis) to strengthen the robustness of patient-reported outcomes [39].

Overall, this study underscores the potential of telehealth to improve primary healthcare accessibility, particularly in managing chronic conditions, reducing healthcare costs, and enhancing patient satisfaction [40]. For telehealth to realise its full potential in Pakistan, targeted efforts are needed to address technological gaps, improve privacy assurances, and strengthen patient-provider communication [41]. These findings contribute to the growing body of evidence supporting telehealth integration into mainstream healthcare systems and provide actionable insights for policymakers and healthcare providers aiming to design patient-centred virtual care models [42].

Equity considerations are critical when evaluating digital health. Younger, urban, and more educated participants were more strongly represented in this sample, suggesting a digital divide that may exclude older adults, rural residents, and those with lower health literacy [43]. Gender differences were not statistically significant in the regression analysis. Yet, the slightly higher female participation indicates that women may already be leveraging telehealth as a more accessible alternative to in-person care [44]. With Punjab and Sindh dominating, regional differences highlight that provinces with weaker infrastructure, such as Balochistan and Gilgit-Baltistan, may face greater barriers to telehealth adoption [15]. Addressing these disparities will ensure equitable scaling of virtual healthcare services. Nearly one-third of respondents expressed dissatisfaction with prescribed medications. As our survey did not capture specific reasons, this finding highlights the need for more structured teleconsultation protocols, including clear dosing instructions, counselling on side effects, and integration with pharmacy support [45].

Gender was recorded as binary (male/female), reflecting the telehealth provider’s database. This excludes non-binary and gender-diverse individuals, who may face unique barriers to telehealth. Future studies should incorporate inclusive gender categories to better reflect diverse patient experiences [46].

Policy frameworks must also address data protection and privacy, provider licensing across jurisdictions, and harmonisation of cross-provincial service delivery. Establishing clear guidelines in these domains will ensure telehealth is safe, equitable, and legally robust [47].

## Conclusion

Telehealth offers a promising solution to bridge healthcare access gaps in Pakistan, where disparities persist due to infrastructure limitations, poverty, and low health literacy. This study highlights high patient satisfaction, strong provider trust, and clear cost savings, particularly in chronic disease management. Patient comfort and perceived care quality emerged as key predictors of telehealth acceptance, underscoring the importance of patient-centred design.

Policy and practice should prioritise strengthening digital infrastructure, improving privacy safeguards, and designing gender- and equity-inclusive strategies to ensure access for older adults, rural populations, and low-literacy groups. Training healthcare providers and enhancing patient education will sustain trust and improve medication management.

Despite limitations related to convenience sampling, self-reported cost data, and underrepresentation of older populations, these findings provide actionable evidence to guide national telehealth policy and integration into primary care. Future research should incorporate provider perspectives and clinical outcomes to build a more comprehensive evidence base for scaling telehealth in Pakistan.

## Electronic supplementary material

Below is the link to the electronic supplementary material.

Supplementary Material 1

## Data Availability

All data generated or analysed during this study are included in this published article.
