# Supplementary material for: Enhancing healthcare access through telehealth: patient-centred insights from Pakistan’s primary care sector
Source: BMC Health Serv Res. 2025 Dec 11;25:1597. doi: 10.1186/s12913-025-13820-4 (PMC12701597; doi:10.1186/s12913-025-13820-4)
Supplement: Supplementary file 1 — Supplementary Material 1 [file 12913_2025_13820_MOESM1_ESM.docx]

**Questionnaire for Research**

**Title of the Study:** To evaluate the effectiveness of telehealth in primary healthcare in Pakistan, focusing on the patient’s perspective.

**Principal Investigator:** Tooba Malik (MSPH Student)

**Affiliation:** Health Services Academy (HSA)**,** Park Road, Chak Shahzad, Islamabad-44000 
Email: [academy@hsa.edu.pk](mailto:academy@hsa.edu.pk)  ,Phone: 051-9255590-4

Patient Name: ________________ Age: ________________ Gender: _______________

Area: ___________________ Contact Number: ________________

How do you review the internet at a digital clinic?

| *Excellent* | *Good* | *Poor* | *Very bad* |  |
| --- | --- | --- | --- | --- |

Do you trust the Doctor's treatment and findings?

| *Very Satisfied* | *Satisfied* | *Somewhat satisfied* | *Dissatisfied* |  |
| --- | --- | --- | --- | --- |

How do you feel about the doctor's behavior?

| *Happy* | *Somewhat happy* | *Okay or Fine* | *Dissatisfied* |  |
| --- | --- | --- | --- | --- |

How do you feel about staff or operator behavior?

| *Happy* | *Somewhat happy* | *Okay or Fine* | *Dissatisfied* |  |
| --- | --- | --- | --- | --- |

What do you think about the telehealth consultation fee?

| *Very Economical* | *Economical* | *Expensive* | *Very Expensive* |  |
| --- | --- | --- | --- | --- |

What is your response about connectivity time?

| *Quick* | *Fine* | *A little more* | *Had to wait more than I had expected* |  |
| --- | --- | --- | --- | --- |

Will you recommend telehealth to your friends and family?

| *Of course* | *Likely* | *Maybe* | *Not Likely* |  |
| --- | --- | --- | --- | --- |

What do you say about medicine usage?

| *Yes* | *No* | *Do not want to* | *Dissatisfied* |  |
| --- | --- | --- | --- | --- |

How do you feel about discussing new symptoms and concerns with your telehealth care provider?

| *Extremely uncomfortable* | *Somewhat uncomfortable* | *Neither comfortable nor uncomfortable* | *Somewhat comfortable* | *Extremely comfortable* |
| --- | --- | --- | --- | --- |

How do you feel about discussing sensitive and personal information with your telehealth care provider?

| *Extremely uncomfortable* | *Somewhat uncomfortable* | *Neither comfortable nor uncomfortable* | *Somewhat comfortable* | *Extremely comfortable* |
| --- | --- | --- | --- | --- |

How do you feel about discussing diagnosis treatment and follow-up recommendations with your telehealth care provider?

| *Extremely uncomfortable* | *Somewhat uncomfortable* | *Neither comfortable nor uncomfortable* | *Somewhat comfortable* | *Extremely comfortable* |
| --- | --- | --- | --- | --- |

Did the telehealth care provider review imaging and Laboratory tests?

| *Extremely uncomfortable* | *Somewhat uncomfortable* | *Neither comfortable nor uncomfortable* | *Somewhat comfortable* | *Extremely comfortable* |
| --- | --- | --- | --- | --- |

Will you undergo an initial clinic visit with a new provider?

| *Extremely uncomfortable* | *Somewhat uncomfortable* | *Neither comfortable nor uncomfortable* | *Somewhat comfortable* | *Extremely comfortable* |
| --- | --- | --- | --- | --- |

Will you undergo an initial clinic visit with a new provider in the presence of your established physician?

| *Extremely uncomfortable* | *Somewhat uncomfortable* | *Neither comfortable nor uncomfortable* | *Somewhat comfortable* | *Extremely comfortable* |
| --- | --- | --- | --- | --- |

Will complete post operative follow up?

| *Extremely uncomfortable* | *Somewhat uncomfortable* | *Neither comfortable nor uncomfortable* | *Somewhat comfortable* | *Extremely comfortable* |
| --- | --- | --- | --- | --- |

Are you confident that communication with your telehealth care provider is secure and private?

| *Extremely uncomfortable* | *Somewhat uncomfortable* | *Neither comfortable nor uncomfortable* | *Somewhat comfortable* | *Extremely comfortable* |
| --- | --- | --- | --- | --- |

What is your age?

| *18-25* | *26-35* | *36-50* | *51-79* |  |
| --- | --- | --- | --- | --- |

What is your education?

| *High school*  *or less* |  | *Diploma or higher* |  |  |
| --- | --- | --- | --- | --- |

What is your means of transport?

| *Does not own a car* |  | *Owns a car* |  |  |
| --- | --- | --- | --- | --- |

What is your comfort score with telehealth care service?

| *Low*  *(8-18)* | *Moderate*  *(19-29)* | *High*  *(30-40)* |  |  |
| --- | --- | --- | --- | --- |

Do you have a chronic disease?

| *Yes* |  | *No* |  |  |
| --- | --- | --- | --- | --- |

Which type of chronic disease do you have?

| *Diabetes* | *Hypertension* | *Asthma* | *Heart Disease* | *Hypothyroidism*    *Other* |
| --- | --- | --- | --- | --- |

Which best describes your physical state or health?

| *My health makes it impossible for me to engage in most activities* | *My health makes it impossible for me to engage in some activities* | *My health makes it difficult for me to engage in some activities* | *I am able to go about my daily activities with minimal difficulty* | *Fully active without restriction Comfort with technology: ease of use Strongly agree or agree* |
| --- | --- | --- | --- | --- |

Are you comfortable with technology and its ease of use?

| *Strongly agree*  *Or agree* |  | *Strongly disagree*  *Or disagree* |  |  |
| --- | --- | --- | --- | --- |

What do you say about this statement? I believe I can get the same quality of care from a video call as from an in-person visit.

| *Strongly agree*  *Or agree* |  | *Strongly disagree*  *Or disagree* |  |  |
| --- | --- | --- | --- | --- |
